# The role of household labour for sustainable intensification in smallholder systems: a case study in cocoa farming systems

**DOI:** 10.1007/s10113-024-02243-2

**Published:** 2024-05-17

**Authors:** Lina M. Tennhardt, Gianna A. Lazzarini, Christian Schader, Kagimu Martin, Eric F. Lambin

**Affiliations:** 1 Research Institute of Organic Agriculture (FiBL), Ackerstrasse 113, 5070 Frick, Switzerland; 2 Earth and Life Institute, University of Louvain, Louvain-la-Neuve, Belgium; 3 Farmer Action Learning for Sustainable Agricultural Management (FALSAM), Kampala, Uganda; 4 Stanford Doerr School of Sustainability, Stanford University, Stanford, CA USA

**Keywords:** Uganda, Cocoa, Labour availability, Sustainable agricultural intensification, Pest and disease management, COVID-19

## Abstract

Sustainable agricultural intensification aims at increasing yields on existing agricultural land without negative environmental impacts. Managing pests and diseases contributes to increasing yields. Without synthetic pesticides, this management is labour intensive. Smallholder farming systems heavily rely on manual and household labour, which will be affected by future demographic changes. Knowledge on how these changes will affect sustainable intensification is limited. Based on a case study of Ugandan cocoa farms, we tested the impact of increased household labour availability on pest and disease management (PDM) practices and pesticide use. We made use of a unique quasi-experimental design, in which household labour increased during the national COVID-19 lockdowns as children did not attend school and family members returned from cities. Our interview data from 2019 to 2021 showed that household labour availability increased on average by 0.8 (±2.5) household members and 16% of labour days per hectare. Using different regression models complemented with qualitative insights, we found that the uptake of alternative PDM practices significantly reduced pesticide quantities and expenditures. The implementation of alternative PDM practices was only weakly influenced by household labour availability and increased with farmer training and trust in alternative practices. These results imply that alternative PDM practices are an important pillar for production with little or without synthetic pesticides and their adoption requires support and incentives, especially on labour or resource-constrained farms.

**Supplementary Information:**

The online version contains supplementary material available at 10.1007/s10113-024-02243-2.

## Introduction

Sustainability has become a guiding principle for global development ambitions (UN 2015) and sustainable agricultural intensification (SI) a leading strategy for agricultural development (Pretty 2018; Rasmussen et al. 2018; Rockström et al. 2017). SI aims at increasing yields to feed humanity without generating adverse environmental impacts or cultivating more land, while also contributing to mitigate major global challenges (Campanhola et al. 2019). Past definitions of intensification in agriculture have prioritised productivity outcomes over environmental or social sustainability (Pretty 2018). SI leverages sustainability for productivity enhancements, which requires a greater capitalisation of ecological processes in agro-ecosystems (Rockström et al. 2017). To achieve this globally across farming systems, SI does not predetermine a selection of agricultural practices or technologies (Pretty 2018) as long as the outcome contributes to multifunctional and sustainable agro-ecosystems that are also sustained by nature (Tittonell 2014).

Pests and diseases represent a major challenge for SI, as they are responsible for up to 30% of yield losses among major food crops (Savary et al. 2019). To control pests and diseases, many farmers resort to synthetic pesticides, of which several have been found to harm human and environmental health (Mahmood et al. 2016; Rani et al. 2021). Pest and disease management (PDM) that involves highly efficient or no application of synthetic pesticides, paired with alternative management practices, can thus be one key pathway towards SI (Pretty 2018). We thus consider alternative pest and disease management (APDM) to be important for SI, especially in smallholder production systems in low- and middle-income countries that produce up to 34% of the global food supply (Ricciardi et al. 2018). These systems face many barriers to increasing yields, such as low nutrient input or lack of investment opportunities (Sheahan & Barrett 2017).

SI practices in smallholder systems with low mechanisation are often labour-intensive (Dahlin & Rusinamhodzi 2019; Leonardo et al. 2015). Mozzato et al. (2018) found that higher family labour availability positively influences environmental practice adoption. The relationship between labour availability and practice adoption is especially pronounced when labour availability is low and the practice is labour saving (Arslan et al. 2020; de Oca Munguia & Llewellyn 2020). Labour availability thus clearly influences smallholders’ decision to invest in SI (Adolph et al. 2020). However, demographic changes, such as urbanisation or population increase, are observed globally. So far, we have only limited knowledge about how these changes will influence SI (Vanlauwe et al. 2014) because conducting experiments on the effect of changing labour availability is difficult.

One exemplary smallholder crop with low levels of mechanisation and strong reliance on manual labour is cocoa (Voora et al. 2019). Pests and diseases are a major cause for low cocoa yields (Kagorora et al. 2021) and their management represents a major challenge for cocoa producers. This has led to agricultural policies in producing countries that promote synthetic pesticides (Aneani et al. 2012). Many cocoa farmers, however, lack knowledge about active ingredients and correct dosage, and lack personal protective equipment for safe application (Oyekale 2018). Simultaneously, the demand for organically produced cocoa has been rising (Meier et al. 2020). Additionally, many chocolate manufacturers increasingly source cocoa with voluntary sustainability certification or through their in-house sustainability programmes. These aim to eliminate (in case of organic certification) or reduce the use of (highly toxic) synthetic pesticides and promote SI and APDM. Importing countries are also regulating pesticide residues in agricultural imports, such as the European Union (The European Parliament 2005). However, many cocoa farmers still depend heavily on synthetic products for pest and disease control in cocoa, generating social and environmental issues (Miyittah et al. 2022).

Many of the APDM practices in cocoa are labour-intensive. Weeds can serve as hosts for pests and diseases and manual weeding requires higher labour investments than spraying herbicides (Bymolt et al. 2018). Labour input for cocoa production that includes pruning, weeding, and phytosanitary measures is greater than for traditional cocoa management (Curry et al. 2015; Folefack et al. 2021; Juhrbandt et al. 2010; Scudder et al. 2022). Diverse agroforestry systems that rely on natural cycles for PDM require greater labour investments than full-sun cocoa systems, mainly due to cocoa and shade tree pruning (Armengot et al. 2016). The amount of available labour can thus determine how smallholder farmers manage their cocoa (Bymolt et al. 2018). Household labour remains an important input for cocoa production as hired labour is expensive (Fountain & Huetz-Adams 2020; Vigneri et al. 2016). Demographic changes also affect cocoa farmers, who increasingly worry about labour shortage (Dormon et al. 2004; Mithöfer et al. 2017). Especially elderly farmers might be unable to keep up proper crop management themselves or hire workers (Abdulai et al. 2020; Kongor et al. 2018). Labour shortage could thus be one hindering factor for farmers to implement APDM (Miyittah et al. 2022).

Past literature on practice adoption in cocoa has looked into farm labour as one explanatory factor among many and has compared labour availability between farms at a single point in time. Quantitative labour data collection is rare and household size is often used as a proxy for farm labour or inputs are estimated by key informants. Studies in Ghana did not find significant relationships between household size and weeding frequency (Aneani et al. 2012), pesticide use (Denkyirah et al. 2017; Kehinde & Adeyemo 2017; Wongnaa & Babu 2020), or pruning and mulching (Wongnaa & Babu 2020). By contrast, Danso-Abbeam et al. (2014) found that larger households in Ghana had lower agrochemicals expenditures for cocoa. Scudder et al. (2022) found a positive relationship between labour investment and improved PDM in Indonesia based on ‘theoretical’ farms. Vigneri et al. (2016) found a general correlation between cocoa yields and adult household labour demand in Ghana and Côte d’Ivoire. To our knowledge, no study has yet specifically assessed how household labour availability affects specific cocoa farming practices, such as PDM.

We aim to contribute to closing this gap by analysing the relationship between household labour availability and pesticide use among smallholder farmers, using smallholder Ugandan cocoa farmers as a case study. Specifically, we analyse whether increased household labour availability, mediated by the implementation of APDM practices, reduces synthetic pesticide use quantities and expenditures. Manipulating household labour availability in an experimental setting is difficult and we thus make use of a unique quasi-experimental design. Following COVID-19 regulation in Uganda, farm labour availability increased due to children not visiting school and family members returning to farming households. We compare data from just before the pandemic and 2 years into the pandemic. Our research questions are as follows: (1) How is additional household labour allocated on smallholder cocoa farms?; (2) Is the implementation of alternative pest and disease management associated with a reduction in pesticide use?; and (3) Do changes in household labour availability increase the adoption of alternative pest and disease management practices in cocoa? Our underlying hypothesis is that, with increasing household labour availability, the share of farms implementing APDM practices increases and the use of synthetic pesticides reduces. We do not expect COVID-like pandemics to occur at a high frequency, but consider the knowledge gained by studying this situation as scientifically important. We followed a quantitative approach, complemented with detailed qualitative insights.

The rest of this manuscript describes the case study setting before presenting the methodological approach. The results show how greater labour availability is invested on farms and how this relates to pesticide use and APDM. We then discuss the results first within our specific case study and then in light of broader SI ambitions on smallholder farms, before providing a conclusion.

## Materials and methods

### Case study description

Cocoa production in Uganda has been increasing (FAO 2022). This is expected to continue due to the countrys increasing climatic suitability for cocoa cultivation (Bunn et al. 2017) and governmental promotion efforts (Kagorora et al. 2021). With 0.494 tons/hectare, average cocoa yields remain lower in Uganda compared to other major cocoa-producing countries, such as Ghana (0.552 tons/hectare) (FAO 2022). Pests and diseases substantially impact yields in Uganda. Farmers primarily rely on synthetic pesticides for pest and disease control (Andersson & Isgren 2021), as they are easily accessible and perceived as effective. Store-bought organic alternatives are not easily available. The widespread use of unregulated or counterfeit pesticides has become a public concern in Uganda (Yiga 2022).

The COVID-19 pandemic in Uganda was met with strict governmental responses. It imposed two major national lockdowns (24.03.-30.04.2020 and 10.06.-30.07.2021) including the closure of schools, suspension of public transport, ban of inter- and intra-district movement, and a night curfew. Schools remained closed from March 2020 to January 2022. Additionally, formal and informal income-generating activities in cities reduced drastically (Kansiime et al. 2021; Steverding & Margini 2020), resulting in the return of many urban workers to their home villages.

Farmers within this case study are located in Mukono district, Central Uganda. They belong to the future supplier base of a Swiss chocolate manufacturer and have been converting to organic certification since 2017, with a first external audit in 2021. A local export company organises the certification process for approximately 450 cocoa and vanilla farmers, including internal control systems and farmer trainings (see Tennhardt et al. (2022) for more details).

### Farmer sampling and data collection

Primary data were collected by two of the authors with a local data collection team within a larger research project on farm-level sustainability. Participating farmers represent a simple random sample of 204 farmers from the above-mentioned group in Mukono district, as this sample size was feasible within the project. Baseline data for the reference year 2019 were collected in February–March 2020 and included a structured interview with farm visit focussing on farm management, sustainability indicators, and contextual information. A second interview with the sample farmers took place in February–March 2022, representing endline data for the reference year 2021. This interview focused on PDM and farm labour characteristics. Participation in this study was voluntary and we obtained oral informed consent from all respondents for the collection and processing of their personal data, documented with participants’ signature in a participation list. Farmers who no longer wanted to participate in the survey and farms with a changed manager were excluded from the analysis, resulting in a final sample of 194 farms.

### Collected data

To test the relationship between farm labour availability and pesticide use, we collected data for three groups of indicators.

#### Pest and disease management practices

Respondents were asked about the synthetic, organic, and home-made pesticides they applied (commercial name, quantities, expenditures in Ugandan shilling (UGX), and ingredients for home-made concoctions^1^) and the crops on which they applied them at both baseline and endline. To reduce recall bias, respondents were asked about the packaging to corroborate quantities and commercial names. We then collected information about active ingredients and their concentrations for each synthetic pesticide mentioned, and calculated quantities of active ingredients (in grammes) for each farm to achieve comparable input quantities. A clear allocation of inputs to individual crops was not possible due to the existence of mixed crops (e.g. agroforestry systems in which farmers sprayed both cocoa and banana without clear knowledge about the share sprayed on each crop). Thus, quantitative information is only available at farm level. Additionally, respondents were asked whether or not they carried out APDM, specifically pruning, applying concoctions, and phytosanitary measures (selectively eliminating diseased pods and plant parts and removing them from the field) in cocoa.

#### Farm labour availability

At baseline and endline, respondents were asked the number of working weeks and the average number of working hours per week for each farm worker. This included household members and hired labour. Individual remuneration status was also asked: none, in-kind, monetary. Remunerated household labour was negligible and we thus calculated the total unpaid household labour and remunerated hired labour hours for the entire farm operation and each reference year, converted into 6-h labour days. By collecting data during the same months at baseline and endline, we avoided some potential bias due to changes in labour needs throughout the year. A household was defined as the unit of people living, eating, and operating a farm together. We collected quantitative information for the entire farm and did not break down labour investments for specific farm activities, as we estimated a high level of uncertainty. Instead, we opted for a more qualitative approach to explore changes in farm labour availability and farm labour allocation to specific tasks during the endline survey. Farmers were asked to estimate how the total time investment for specific cocoa-growing activities changed since the start of the COVID-19 pandemic in comparison to before the pandemic (response options: increased, similar, decreased, practice not done on the farm).

#### Control variables

To control for different factors that might influence pesticide use and expenditures on cocoa farms, we collected numerous additional variables for each farm at both baseline and endline. These included farmer factors, comprising farm managers’ age, gender, farming experience, household size, training participation, and seven pesticide need statements with a 5-point Likert scale as response option with which we calculated a pesticide need index (mean value; Supplementary material 4). This was used as a proxy for farmers trust in APDM practices. We furthermore collected data on farm characteristics, i.e. farm size and revenues from single crops and products.

## Statistical analyses

### Comparative analyses

For an overview of general changes among sampled farmers, we compared key farm characteristics of the entire sample between 2019 and 2021. We then grouped farms into two groups based on changes in household labour availability: those with increased availability and those with similar or lower availability. We examined the differences in time investment for specific cocoa growing activities before COVID-19 to 2021, along with other control variables between the subgroups (e.g. farm size, cocoa yields, cocoa revenues). We compared categorical variables using chi-squared test and non-parametric continuous variables using Wilcoxon rank sum test, applying paired tests for the comparison of same farms in different years.

### Causal identification

We hypothesised that increases in household labour availability reduce the amount of synthetic pesticides applied on farms because labour-intensive APDM would be carried out instead. We tested this hypothesis in a two-step approach. First, we analysed the influence of the APDM practices pruning, phytosanitary practices, and concoctions use in cocoa on pesticide quantities and expenditures. Second, we analysed the influence of increased household labour availability on the implementation of these practices. We estimated ordinary least square (OLS) regression and generalised linear regressions (GLM) models of our cross-sectional data and fixed effects (FE) models using our panel data. This combination of methods was selected for robustness check and to exploit the strength of each methodology: OLS and GLM allowed us to control for time-invariant factors and coefficients for which data from only 1 year were available, while they do not allow for causal inferences. FE models, on the contrary, allowed us to control for both unobserved time-invariant farm level variables that might affect the outcome variables as well as time-variant unobserved variables that may affect all farms equally.

- **a. The effect of alternative pest and disease management practices on pesticide quantities and expenditures**

#### Ordinary least square (OLS) models

We estimated OLS models to test the relationship between changes in pesticide quantities and expenditures and the implementation of APDM practices. All data refer to the entire farm size. To control for other factors that might influence the outcome variables, we added a vector of covariates from the 2019 data with few exceptions from the 2021 data (Table 3). These included farm-specific covariates (farm size, commercial vegetable production (dummy), average pesticide prices per kg of active ingredient, and remittances (dummy)) and farmer-specific covariates (gender, age, farming experience, years of formal education, household size, training participation since 2020 (dummy), and a pesticide need index).

We estimated an OLS model (Eq. 1) in which $\Delta {Y}_{i}$ represents the change in outcome variable, i.e. pesticide quantities and expenditures, by subtracting 2019 from 2021 data at the individual farm-level $i$. ${\beta }_{0}$ represents the intercept for sampled farms and ${\beta }_{1}$ the population slope coefficient for ${X}_{i}$, a vector of the three dichotomous alternative practices for the $i$ th individual farm. ${\beta }_{2}$ represents the population slope coefficient for ${E}_{i}$, representing a vector of variables that potentially influence the outcome variable. ${\varepsilon }_{i}$ represents a random error term. Our main interest lies in the coefficient ${\beta }_{1}$ as it indicates the relationship between changes in pesticide quantities and expenditures and the implementation of alternative practices.

1 $$\Delta {Y}_{i} = {\beta }_{0}+ {\beta }_{1}{X}_{i}+ {\beta }_{2}{E}_{i }+ {\varepsilon }_{i}$$

The distribution of the dependent variables was continuous, including negative, zero, and positive values, with a wide distribution. To retain negative and zero values, we applied an inverse hyperbolic sine (IHS) transformation (Bellemare & Wichman 2020). Our models show a predominantly linear relationship between the residuals and fitted values, with the exception of three outliers. We thus assume the linearity assumption to be satisfied. We verified the non-existence of multicollinearity and correlation with the error term in our models using variance inflation factors (all values <2). The distribution of residuals across the models shows some deviation from normality, indicating potential violations of the OLS assumptions of random sampling and a conditional mean of zero. Furthermore, our models suffer from slight heteroskedasticity. For completeness, we have also included an OLS regression for the direct relationship between changes in synthetic pesticides and changes in household labour availability (Supplementary material 6).

#### Linear two-way fixed effects (FE) models

Making use of the two-period balanced panel data, we used a FE model to gain additional insights into how changes within each farming household influenced the outcome variables, i.e. pesticide quantities and expenditures. We introduced both a farm and a year FE.

We estimated the linear FE model using Eq. 2, in which ${Y}_{it}$ represents the outcome variable for an individual farm $i$ at time $t$. ${\beta }_{i}$ represents farm-specific intercepts that capture heterogeneities across farms and ${\beta }_{t}$ represents time-specific intercepts that capture heterogeneities over time. ${\beta }_{1}$ represents the coefficient of the vector of variables of interest ${X}_{it}$, i.e. the implementation of APDM practices, and measures the causal effect on ${Y}_{it}$. We additionally control for time-varying confounders in vector ${F}_{it}$ and included training participation since 2020 (dummy), commercial vegetable production (dummy), and remittances (dummy). ${Z}_{it}$ represents a vector of time-invariant explanatory variables and ${\varepsilon }_{it}$ a random error term for an individual farm $i$ at time $t$. After estimating the FE model, the time-invariant vector ${Z}_{it}$ cancels. We clustered the standard errors at farm-level to control for heteroskedasticity and autocorrelation.

2 $${Y}_{it} = {\beta }_{i}+ {\beta }_{t}+ {\beta }_{1}{X}_{it}+ {\beta }_{2}{F}_{it}+ {\beta }_{3}{Z}_{it}+ {\varepsilon }_{it}$$

- **b. The effect of changes in labour availability on use of alternative pest and disease management practices**

#### Generalised linear models (GLM)

We estimated GLM models to test the relationship between the dichotomous outcome variables, i.e. implementation of APDM practices, and changes in household and hired labour availability, subtracting 2019 from 2021 data. Similar to the OLS models, we considered additional covariates (Table 4) that could affect practice implementation on farms. The GLM models also included household size and households’ dependency ratio, i.e. the share of household members <15 and >64 years.

We estimated a logit model, in which the dichotomous outcome variable ${Y}_{i}$ with {0,1} indicates whether or not a practice is implemented on an individual farm $i$, and $p$ indicates the probability of $Y$ = 1, *p* = *P*($Y$ = 1). $\Delta {X}_{i}$ in Eq. 3 represents the vector of the predictor variables of interest, i.e. changes in household and hired labour availability between 2019 and 2021 and ${E}_{i}$ represents a vector of additional covariates that might influence the outcome variables. The logistic regression estimates ${\beta }_{0}$, the intercept for sampled farms, as well as ${\beta }_{1}$ and ${\beta }_{2}$, representing the respective population slope coefficient for the vectors $X$ and $E$, via maximum likelihood method:

3 $${\text{logit}}\left(p\right)= \mathit{log}(p /(1-p))={\beta }_{0}+ \Delta {\beta }_{1}{X}_{i}+ {\beta }_{2}{E}_{i}$$

#### Conditional fixed effects (FE) logit models

The conditional FE models were estimated to test the influence of household and hired labour availability and the dichotomous outcome variables describing the implementation of APDM practices. Similar to the linear FE models, we introduced both a farm and a year FE to the models and controlled for other time-varying confounders.

We employed logit FE model to examine the likelihood of a farm to implement individual APDM practices ${Y}_{it}$ with {0,1}, where $i$ and $t$ index farms and years. As FE models examine change over time, the case of interest is ${Y}_{i1}+ {Y}_{i2} =1$, which can either be ${w}_{i}=1$ when ${(Y}_{i1},{Y}_{i2}) =(\mathrm{0,1})$ or ${w}_{i}=0$ when ${(Y}_{i1},{Y}_{i2}) =(\mathrm{1,0})$. All farms without change in the outcome variable and thus ${Y}_{i1}+ {Y}_{i2} \ne 1$ are excluded from the model and do not contribute to the coefficient estimate. Based on Chamberlain (1980), the probability that a farm adopts a practice over time and thus ${w}_{i}=1$ is:

4 $$\mathit{Pr}({Y}_{it}=1 \left| {Y}_{i1}+ {Y}_{i2} =1\right)=\frac{\mathit{Pr}\left({w}_{i}=1\right)}{\mathit{Pr}\left({w}_{i}=0\right)+Pr({w}_{i}=1)}=\frac{EXP(\beta \mathrm{^{\prime}}({X}_{i2}-{X}_{i1})}{1+EXP(\beta \mathrm{^{\prime}}({X}_{i2}-{X}_{i1})}=F[\beta \mathrm{^{\prime}}({X}_{i2}-{X}_{i1})]$$

In Eq. 4, $\beta$ represents the coefficient of the vector of variables of interest ${X}_{it}$, i.e. household and hired labour hours per hectare.

### Software used

The comparative analyses and analyses using Eqs. 1–3 were performed in R (vers. 4.1.0, R Project for Statistical Computing, RRID:SCR_001905), via RStudio (vers. 2022.07.00+548, RStudio, Q19 RRID:SCR_000432). The following R functions and packages were used: From the stats package, the lm() function was used for OLS regressions, the glm() function for the GLM regressions, and the fisher.test() function for Fisher’s exact test. From the fixest package, we used the feols() function for the FE models. The analyses using Eq. 4 were carried out in Stata (StataSE vers 17) using the xtreg command. Data and R-code are available here.

### Methodological limitations

Our data did not distinguish between additional household labour from adults or children, who provide different labour intensities. The return of migrants to the farm also led to lower remittances, potentially influencing farm management. Data quality concerns include uncertainties in estimating farm size, input quantities, and expenditures. This is mostly attributed to the absence of official land titles specifying exact farm sizes, which is a common situation (Vigneri et al. 2016), and the lack of bookkeeping for verifying input quantities and expenditures throughout the reference year. Collecting farm labour data is particularly difficult due to common substantial changes in a ‘normal’ day and a recall bias (Arthi et al. 2018; Bymolt et al. 2018). To reduce this bias, we only considered labour investments for the entire farm and did not quantitatively break them down to specific crops or farming practices. Collecting data identically at baseline and endline ensured a constant bias, thus ensuring comparability. We furthermore included household size as a proxy for household labour with a lower measurement error.

## Results

### Descriptive results

We combine descriptive statistics of our quantitative data with qualitative insights collected during the farmer interviews to set the scene and provide insights into general impacts of the COVID-19 pandemic in the study region.

#### General farm information

General characteristics of the final sample of 194 cocoa farms in Mukono district (Table 1) reveal that farm managers were mainly male and managed on average 2.9 hectares of land in 2019. Farm and cocoa revenues did not change significantly between the years despite farms selling significantly less products in 2021 compared to 2019. Significantly more farmers reported having off-farm income sources in 2021 compared to 2019 (75% and 55%, respectively). Despite cocoa yields increasing significantly from 0.36 to 0.64 tons/hectare, cocoa revenues remained similar. Estimated crop shares of the total farm area indicate that cocoa is on average the crop with the largest cover (17% ±12%) followed by coffee (14% ±11%) (Supplementary material 1). Input-intensive grain and vegetable production cover 11% (±10%) and 10% (±8%) of the farm area on average.

**Table 1:** Mean (sd) and *n* (percentage) of general farmer and farm information for 2019 and 2021

	2019 (*n* = 194)	2021 (*n* = 194)	Difference	*p*-value
Age of farm manager (years)	52.65 (12.51)	54.06 (13.54)	+1.41	< 0.001
Male farm manager (% farmers)	133 (68.6%)	131 (67.5%)	−2	0.789
Formal education farm manager (years)	6.98 (3.57)	6.98 (3.57)	0	1.000
Household size (people)	5.73 (2.72)	6.36 (2.91)	+0.63	< 0.001
Livestock ownership (% farmers)	133 (68.6%)	175 (90.2%)	+42	< 0.001
Farm size (ha)	2.85 (3.02)	2.85 (3.02)	0	1.000
Cocoa yields (tons/ha)	0.36 (0.46)	0.64 (0.67)	+0.28	< 0.001
Cocoa revenue (million UGX)	2.40 (3.65)	2.54 (4.25)	+0.14	0.221
Farm revenue (million UGX)	6.10 (8.45)	5.50 (7.04)	−0.6	0.188
Sold farm products (number)	4.37 (2.29)	3.95 (1.63)	−0.42	0.041
Off-farm income (% farmers)	106 (54.6%)	145 (74.7%)	+39	< 0.001

Statistical comparison between years using paired McNemar’s chi-squared test with continuity correction for binary variables; paired Wilcoxon rank sum test with continuity correction for continuous variables

#### COVID-19 impacts

Farmers reported mainly negative economic impacts through the COVID-19 pandemic and government responses. Prices for agricultural goods destined for national and international markets generally decreased. The products for national markets included vegetables and eggs, which farmers usually sold in towns or to local schools through agreements. Transportation costs increased drastically following COVID-19 regulation, leaving many farmers unable to pay and resorting to sell their produce in the villages, which again led to oversupply and plummeting prices. Cash crops for international markets included both vegetables and spices for neighbouring countries like Kenya, and coffee and cocoa for global markets. Difficulties in border crossings delayed exports and markets for perishable goods ceased. Coffee and cocoa had relatively stable markets and prices. Transportation restrictions led to fewer coffee and cocoa traders, resulting in lower prices and credit-based cocoa purchase with repayments made in instalments to the farmers. Export companies for these crops are often well-established, unlike other products which are distributed by personal trucks. Consequently, most farmers in our sample cited cocoa and coffee as their primary income source during the pandemic.

### Changes in pest and disease management

The share of sampled farmers applying synthetic pesticides on their farm and in cocoa remained similar for 2019 (63% and 40%, respectively) and 2021 (65% and 42%, respectively) (Table 2). This was despite 58% of farmers who perceived that pest and disease pressure in cocoa was lower in 2021 compared to prior years (Supplementary material 3), attributed to more rainfall and thus better cocoa growth. Yet, it is common practice for farmers in Mukono to carry out preventative calendar instead of curative pesticide applications to manage major pests, including green stink bugs (*Bathycoelia thalassina*) and mealy bugs (*Planococcus* spp.). Qualitative information from farmers revealed that they had been avoiding synthetic pesticide application in cocoa as they were undergoing organic certification since 2017 and were promised higher cocoa prices once certified. As their cocoa was still not bought with price premiums at the time of the second data collection, many farmers resorted back to spraying synthetic pesticides out of fear of losing their harvests. Few farmers applied biopesticides (0.5% in 2019 and 1% in 2021), yet qualitative information revealed farmers’ general willingness to buy them if they were effective and available. Only 7% of farmers reported input shortages due to national COVID-19 restrictions (Supplementary material 3).

**Table 2:** Mean (sd) and *n* (percentage) of pest and disease management variables as well as farm labour availability in 2019 and 2021

	2019 (*n* = 194)	2021 (*n* = 194)	Difference	*p*-value
Pesticide use (% farms)	123 (63.4%)	126 (64.9%)	+1.5%	0.798
Pesticide use in cocoa (% farms)	77 (39.7%)	82 (42.3%)	+2.6%	0.614
Pesticide use (kg of active ingredient/ha)^a^	0.86 (2.37)	1.52 (5.15)	+0.66	0.701
Expenditures synth. pesticides (1000 UGX/ha)^a^	44.61 (92.96)	59.90 (123.28)	+15.29	0.052
Active substances applied (#)^a^	1.63 (1.77)	1.89 (2.04)	+0.26	0.040
Expenditures biopesticides (1000 UGX/ha)^a^	0.08 (1.1)5	0.17 (1.82)	+0.09	0.789
Expenditures homemade concoctions (1000 UGX/ha)^a^	0.62 (3.29)	2.74 (9.57)	+2.12	< 0.001
Concoctions use in cocoa (% farms)	45 (23.2%)	75 (38.7%)	+15.5%	< 0.001
Pruning of cocoa trees (% farms)	60 (30.9%)	162 (83.5%)	+52.6%	< 0.001
Phytosanitary measures in cocoa (% farms)	76 (39.2%)	122 (62.9%)	+23.1%	< 0.001
Household labour (labour days/ha)	394 (426)	506 (534)	+112	< 0.001
Hired labour (labour days/ha)	0.09 (0.28)	0.01 (0.02)	−0.08	< 0.001
Total labour (labour days/ha)	474 (431)	519 (536)	+45	0.120
Household members (#)	3.89 (2.36)	4.79 (2.43)	+0.91	< 0.001

Statistical comparison between years using paired McNemar’s chi-squared test with continuity correction for binary variables; paired Wilcoxon rank sum test with continuity correction for continuous variables

^a^Values refer to the entire farm area

The active ingredients most applied by farmers in 2019 and 2021 were the insecticide cypermethrin (43% and 42%, respectively; Supplementary material 2) and the herbicide glyphosate (39% and 26%, respectively). In total, 11 active ingredients were applied by >5% of farmers, six of which are considered ‘Bad actors’^2^ and eight of which are regarded as highly hazardous pesticides.^3^

The share of farms implementing APDM practices increased from 2019 to 2021. Concoctions in cocoa increased from 23 to 39% of sampled farmers (Table 2). Qualitative insights reveal that farmers perceive collecting plants and brewing concoctions to be tedious and not suitable for large plantations. The share of farmers pruning cocoa increased from 31 to 84% and applying phytosanitary measures from 39 to 63%.

### Changes in farm labour availability

Our results show a significant increase in the number of household members involved in farm work from an average of 3.9 in 2019 to 4.8 in 2021 (Table 2). Qualitative information from farmers revealed that family members and friends joined them in their rural homestead when the national lockdowns were announced to avoid income and food shortage in urban areas. Despite 24% of farmers reporting cases of COVID-19 infections within their household, only four farm managers mentioned consequent longer-term absences from farm work. The median number of 6-h working days per hectare invested by household members in farm work increased by 16% from 2019 to 2021. Qualitative insights attribute this growth to additional household members, children out of school, and lower opportunities for off-farm income due to governmental restrictions. Simultaneously, hired labour days/ha reduced significantly from 0.09 to 0.01, and only 19% of farms hired more labour in 2021 than in 2019. Qualitative information suggests that plenty of willing workers were available in the villages at low cost, yet farmers hired less due to economic constraints or no need.

Our sample included a subgroup of 128 farms with more household labour available in 2021 compared to 2019 and a subgroup of 66 farms with similar or less household labour availability. These two subgroups had largely similar characteristics (Supplementary material 5). However, farms with increased household labour availability were more often male-managed and had higher cocoa revenues in 2019 and 2021. The share of farmers applying concoctions in cocoa was significantly higher among farmers with increased household labour availability in 2021.

### Changes in farm labour allocation

Based on qualitative information, farmers reported that most additional household labour was allocated to producing food crops to cater for the increased amount of household members or send food to relatives in cities. According to our respondents, additional household members and children mainly engaged in manual weeding and harvesting, as these practices do not require specific skills.

**Fig. 1:**
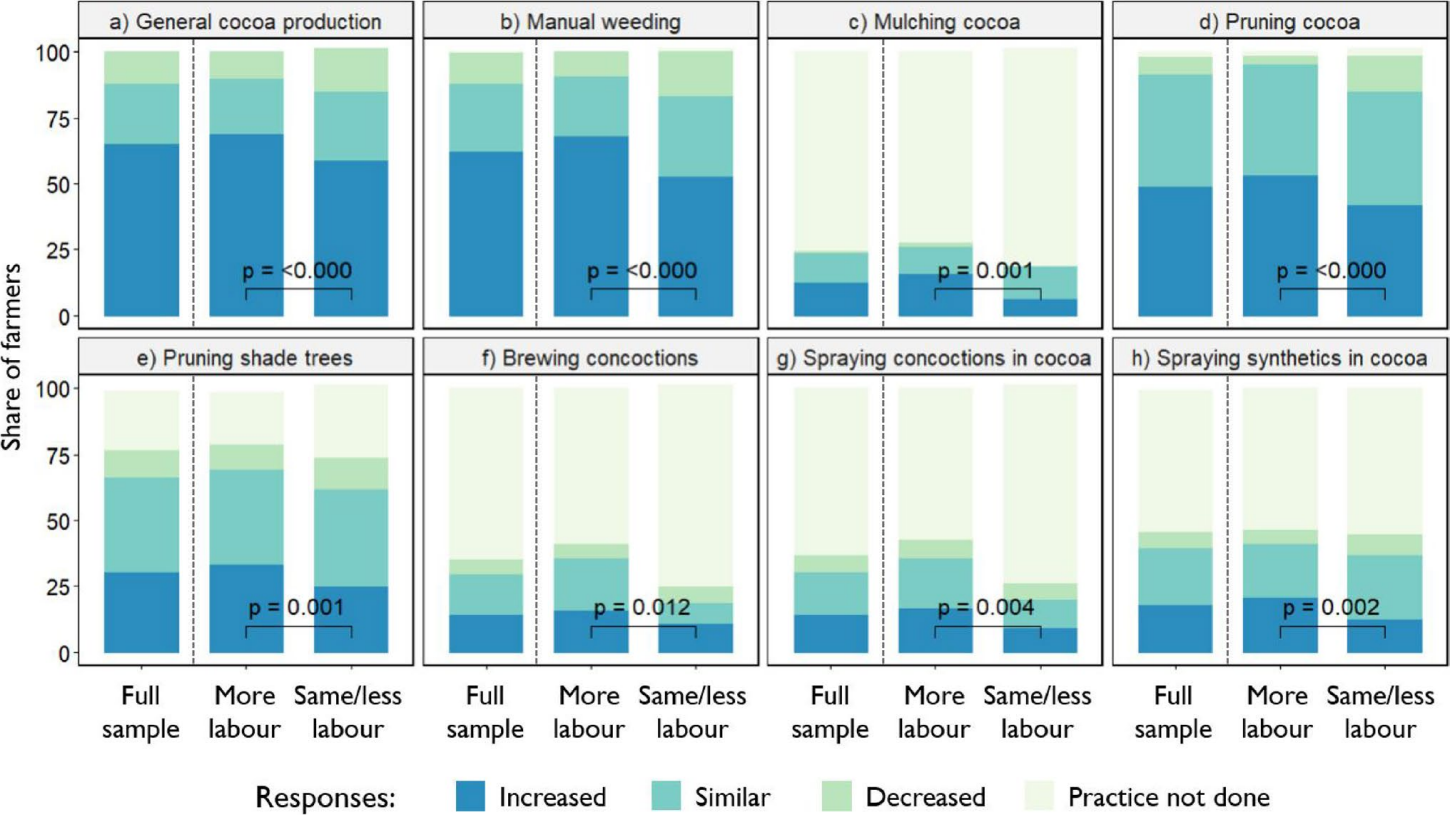
Estimated changes in labour time investment for specific cocoa production tasks in 2021 compared to pre-COVID-19 times for the full farmer sample and the subsamples with and without increased household labour availability; statistical comparison of the response option ‘Increased’ between the two subsamples using a Fisher’s exact test

The majority of farmers (65%) perceived their labour investments in cocoa production in 2021 to be greater than prior to the COVID-19 pandemic (Fig. 1). This additional labour was mainly invested in manual weeding, which increased on 62% of farms, and pruning of cocoa and shade trees, which respectively increased on 49% and 30% of farms. The perceived changes in total labour time investment for specific cocoa growing tasks in 2021 compared to pre-COVID times between the subsamples with and without increased household labour availability. As much as 69% of the subsample with increased household labour availability augmented their time investment in cocoa production compared to 58% of the subsample with similar or lower household labour availability (Fig. 1). This difference is also found when comparing specific practices, like manual weeding and cocoa pruning. A significantly larger number of farms with increased household labour availability augmented time investments in spraying synthetic pesticides compared to farms without increased household labour availability (20% and 12%, respectively). Qualitative information revealed that fetching the water for mixing synthetic pesticides is labour intensive and if outsourced costs 500 UGX for 20 l, discouraging farmers with large fields to spray.

### Impact of increased household labour availability on pesticide use and expenditures

The results of the OLS and FE models testing the influence of APDM practices on pesticide quantities and expenditures are presented in Table 3. The OLS models showed a statistically significant and positive relationship between using concoctions in cocoa and changes in pesticide use quantities and expenditures. This indicates that farms that applied concoctions in cocoa in 2019 increased pesticide quantities and expenditures in 2021 compared to 2019. The FE models, however, showed a significant and negative influence of concoctions use and phytosanitary measures in cocoa on pesticide quantities and expenditures. This suggests that with the uptake of these practices within farms over time, pesticide quantities and expenditures decrease. Furthermore, commercial vegetable production significantly and positively influenced pesticide quantities and expenditures.

**Table 3:** Results of the ordinary least square (OLS) and linear fixed effects (FE) models testing the relationship between practice implementation and pesticide use and expenditures. FE models include farm and year FE

	Pesticide quantities (g active ingredient/ha)^a^		Pesticide expenditures (UGX/ha)^a^	
	Change 2021–2019	2019 and 2021	Change 2021–2019	2019 and 2021
	OLS	FE	OLS	FE
Pruning cocoa (1/0)^b^	−0.33 (0.96)	−0.82 (0.47)	−1.70 (1.52)	−1.39 (0.71)
Concoctions use (1/0)^b^	2.85** (0.93)	−1.97*** (0.56)	3.09* (1.46)	−2.92*** (0.86)
Phytosanitary measures cocoa (1/0)^b^	−0.34 (0.90)	−1.21** (0.40)	1.82 (1.36)	−2.07** (0.65)
Male farm manager (1/0)^b^	−1.24 (0.95)		−0.04 (1.59)	
Age farm manager (yrs)^b^	0.09 (0.05)		0.08 (0.07)	
Education farm manager (yrs)^b^	0.04 (0.12)		0.02 (0.20)	
Farming experience (yrs)^b^	−0.05 (0.04)		−0.08 (0.06)	
Training since 2020 (1/0)	−0.95 (0.83)	0.09 (0.52)	−0.05 (1.33)	0.39 (0.849)
Commercial vegetable production (1/0)^c^	7.19*** (1.70)	1.80* (0.73)	7.91** (2.58)	2.24* (1.10)
Pesticide price (M UGX/kg AI)^c^	−4.97 (2.85)		8.57 (4.71)	
Farm size (ln(ha))	−0.41 (0.56)		−0.85 (0.91)	
Pesticide need index^c^	2.32*** (0.40)		3.92*** (0.63)	
Remittances (1/0)^c^	−1.79 (1.58)	−1.50* (0.61)	−2.53 (2.40)	−2.29* (1.03)
Constant	−8.51*** (2.57)		−12.73*** (3.74)	
Observations	194	388	194	388
*R*^2^	0.27	0.747	0.27	0.723
Adjusted *R*^2^	0.21	0.476	0.22	0.427
Residual Std. Error (df = 179)	5.65		8.8	
*R*^2^ within		0.173		0.160
*R*^2^ within Adj.		0.147		0.133
*F* statistic (df = 14; 179)	4.68***		4.83***	

*p<0.05; **p<0.01; ***p<0.001, robust standard errors in OLS and clustered standard errors in FE

^a^IHS transformed

^b^2019 data in OLS regressions

^c^2021 data in OLS regressions

The results of the GLM and FE models testing the influence of increased household labour availability on APDM practices are presented in Table 4. The GLM models showed a significant and positive relationship of increased household labour availability and concoctions use in cocoa. The FE models did not mirror these results. They showed a significant and positive influence of household members, as a proxy for household labour availability, on cocoa pruning. Yet, an increasing dependency ratio significantly and negatively influenced cocoa pruning. The GLM models furthermore showed a significant and negative relationship of concoctions use and phytosanitary measures in cocoa and farmers’ perceived pesticide need. Finally, the FE models showed that training participation since 2020 significantly increased APDM within farms over time. Although the effect is weak, these results suggest that increased household labour availability slightly influenced the implementation of APDM practices within our sample.

**Table 4:** Results of the generalised linear (GLM) and conditional fixed effects (FE) logistic models testing the relationship between household labour availability and alternative practice implementation. FE models include farm and year FE

	Pruning cocoa		Concoctions use in cocoa		Phytosanitary measures in cocoa	
	2021	2019 and 2021	2021	2019 and 2021	2021	2019 and 2021
	GLM	FE	GLM	FE	GLM	FE
Household labour (100 h/ha)^a,d^	−0.04 (0.07)	−0.009 (0.008)	0.14* (0.07)	0.006 (0.012)	0.10 (0.06)	0.005 (0.010)
Hired labour (100 h/ha)^a,d^	0.01 (0.15)	−0.058 (0.04)	−0.02 (0.12)	−0.020 (0.025)	−0.06 (0.11)	−0.051 (0.035)
Household size (# people)^d^	0.10 (0.16)	0.604** (0.215)	−0.02 (0.12)	−0.225 (0.318)	0.19 (0.12)	0.398 (0.235)
Dependency ratio (% household members)^c^	0.01 (0.01)	−0.037* (0.017)	0.005 (0.01)	0.005 (0.019)	0.002 (0.01)	0.001 (0.018)
Training since 2020 (1/0)	0.09 (0.44)	2.251*** (0.516)	0.64 (0.37)	1.023* (0.448)	0.11 (0.34)	0.814* (0.366)
Male farm manager (1/0)^b^	−0.29 (0.50)		−0.07 (0.41)		0.02 (0.40)	
Age farm manager (yrs)^b^	−0.03 (0.06)		0.01 (0.02)		−0.001 (0.02)	
Education farm manager (yrs)^b^	0.04 (0.06)		0.003 (0.05)		−0.03 (0.05)	
Farming experience (yrs)^b^	0.03 (0.02)		−0.01 (0.02)		−0.01 (0.02)	
Farm size (ln(ha))	0.30 (0.28)		0.41 (0.26)		0.08 (0.22)	
Importance cocoa (% farm revenues)^d^	0.07 (0.60)	1.737 (0.965)	0.18 (0.58)	0.164 (1.029)	−0.14 (0.56)	−0.107 (0.849)
Pesticide need index^c^	−0.37 (0.22)		−1.11*** (0.22)		−0.45* (0.18)	
Constant	2.45 (1.38)		1.21 (1.26)		1.99 (1.06)	
Observations	189	234	189	120	189	176
Nagelkerke pseudo-*R*^2^	0.095		0.321		0.112	
Log likelihood	−80.42	−44.99	−101.02	−36.60	−115.97	−48.97
AIC	186.85		228.04		257.94	

*p<0.05; **p<0.01; ***p<0.001, robust standard errors in GLM

^a^IHS transformed in GLM regressions

^b^2019 data in GLM regressions

^c^2021 data in GLM regressions

^d^2021–2019 data in GLM regressions

## Discussion

### How is additional household labour allocated on smallholder cocoa farms?

Increasing household sizes appear to be a two-sided coin for smallholder households. They require higher expenditures and infrastructure, which can place a burden on limited farm incomes. Yet, they also represent seemingly free farm labour, which is highly important in systems with limited mechanisation, heavily depending on manual labour (Dahlin & Rusinamhodzi 2019). In our sample, increased household labour was first used to cover the basic household needs for food. Once covered, additional investments were made in other (cash) crops. However, our data did not show a correlation between household labour investment and farm revenues or cocoa yields, contrasting Juhrbandt et al. (2010) and Higuchi et al. (2022).

As household labour increased, farms in our sample hired less labour. Our data are not conclusive on whether these savings were fully allocated to larger household expenditures. Farms typically hire labour when household labour is constrained or they have strong incentives to invest in certain practices (Bymolt et al. 2018; Martínez & Martínez Pachón, 2021). Literature shows that up to 37% of cocoa farm expenditures are invested in hired labour (Folefack et al. 2021). Increasing ‘free’ household labour might free up some economic resources for other purposes.

### Is the implementation of alternative pest and disease management associated with a reduction in pesticide use?

In our sample, 63% of farmers applied synthetic pesticides in cocoa in 2019. This number is higher than the 10% reported from fully certified organic cocoa farmers in Ghana (Awudzi et al. 2022). Yet with a median application of 0.15 kg of active ingredient/ha in 2019, farmers in our sample used less synthetic pesticides than conventional cocoa farmers in Ghana with 0.47 kg/ha (Schader et al. 2021). Our quantitative data confirmed that the implementation of certain APDM practices can reduce pesticide quantities and expenditures on cocoa farms. Cocoa pruning did not show the same results as concoctions and phytosanitary measures. Apparently, farmers perceived concoctions and phytosanitary measures as actual alternatives to synthetic pesticides. Most farmers in our sample applied prophylactic calendar spraying, potentially concealing beneficial effects of APDM on pest and disease pressure. Transitioning from prophylactic to curative spraying is challenging and requires great trust in alternative practices and inputs. Perceived risks can delay practice adoption, especially among farmers with fewer economic resources (Foster & Rosenzweig 2010). Changing calendar spraying also depends on seasonal climatic changes and local agro-dealers, who recommend farmers when and what to spray.

Organic certification bans synthetic pesticides and might influence their use in our sample. Most farmers were willing to comply with organic regulation, yet lost motivation over time. Many revert to spraying synthetic pesticides to secure their yields, especially after their cocoa was not bought with a price premium, even after 5 years of conversion. Promising premiums for compliance and not following through represents a major breach of trust and commitment from export companies, as it impacts the income of resource-poor farmers. Trust and commitment are key relationship elements to ensure compliance with sustainability goals (Kumar & Rahman 2015). Yet even farms that are fully organic certified do not always comply with organic regulation, as shown for Fairtrade-organic certified coffee farms in Uganda (Vanderhaegen et al. 2018) or organic certified cocoa farmers in Ghana (Awudzi et al. 2022; Schader et al. 2021).

Cocoa revenues represented on average 39% (2019) and 45% (2021) of farm revenues in our sample. Other input-intensive crops were also of economic importance for farmers. Especially commercial vegetable production increased pesticide use in our sample, common in small-scale vegetable production (De Costa et al. 2021; Enthoven & Van den Broeck 2021; Mergia et al. 2021). Organic certification of small farms does not require a whole-farm conversion. A risk for human and environmental health persists if other plots are still treated with synthetic pesticides. Drift can easily transport pesticide residues to cocoa plots (Benzing et al. 2021). If the aim is to reduce pesticide residues in cocoa, the entire farming system might need to be addressed.

### Do changes in household labour availability increase the adoption of alternative pest and disease management practices in cocoa?

Our qualitative information showed that perceived time investments in cocoa management and APDM increased significantly more often on farms with increased household labour availability. Our quantitative results, however, only allow for a very cautious conclusion that increased household labour availability positively influences adoption of APDM. The significant positive influence of household members on cocoa pruning and significant negative influence of households’ dependency ratio on pruning suggests that pruning requires a strong workforce (Andres et al. 2016).

APDM increased significantly for the entire sample, illustrating a general willingness of farmers to test or implement different PDM. This might have been motivated externally by organic certification, or intrinsically, as most farmers know and have experienced the negative effects of synthetic pesticides on human and environmental health. This is in line with Andersson and Isgren (2021) and Miyittah et al. (2022). However, farmers fear that not spraying their crops will reduce their yields and ultimately their livelihoods. Spraying effective organic pesticides was mentioned as an alternative option; however, they are rarely sold in local agro-shops. This is a major reason for non-compliance with organic regulation among certified cocoa farmers in Ghana (Awudzi et al. 2022). The adoption of PDM practices might also be influenced by farmers’ economic concerns and the question whether increased dedication to cocoa production pays off. While Juhrbandt et al. (2010) and Bymolt et al. (2018) found that investing labour in good cocoa management is economically beneficial, Scudder et al. (2022) found that improved cocoa management required more labour input and was economically not viable for farmers at current cocoa prices.

Two years into the COVID-19 pandemic, schools reopened and life in Ugandan towns and cities was almost back to normal. This motivated many people to return to their urban jobs, thus changing household labour availability again. It remains to be seen whether farmers return to hiring more labour, which labour-intensive practices they will prioritise given lower household labour availability, and whether APDM will become established.

### The role of labour availability for sustainable intensification of labour-intensive cash crops in smallholder systems

While our results only allow for cautious conclusions that increased household labour availability may promote APDM as important practices for SI in smallholder systems, past research has generated stronger claims. Access to resources and labour is often mentioned as one or the major barrier for SI in smallholder farming systems in Sub-Saharan Africa (Adolph et al. 2020; Dahlin & Rusinamhodzi 2019; Kuyah et al. 2019; Tittonell & Giller 2013), including cocoa (Abdulai et al. 2020). Limited labour availability can motivate farmers to use their time better and increase eco-efficiency (Heidenreich et al. 2022). Yet when household labour availability shrinks below what is needed, hired labour needs to fill the gap. Following this logic, the most resource-constrained farmers are less likely to implement practices for SI (Martin et al. 2018; van Vliet et al. 2021) and remain stuck in a ‘poverty trap’ (Tittonell & Giller 2013). Sustainable sourcing practices, which are now common in cocoa value chains, might exacerbate this. Better-off farmers are more likely to participate in sustainability certification of cash crops (Dietz et al. 2021; Jones & Gibbon 2011) and sustainability initiatives tend to focus on larger farms for efficiency reasons (Hirons et al. 2018).

Household labour investments in SI are also linked to opportunity costs: farmers with low opportunity costs are more likely to adopt sustainable practices (Piñeiro et al. 2020; Wollni & Andersson 2014). Diverse demographic trends have different implications for opportunity costs (Chiarella et al. 2023). With increasing urbanisation and young people leaving farming, household labour availability will likely diminish and raise the cost of hired labour (Bymolt et al. 2018; Mithöfer et al. 2017). Consequently, farmers’ opportunity cost to invest their labour on their own farm increases and the attractiveness of earning off-farm income as labourers may rise. Akoyi and Maertens (2018) calculated a daily revenue of 8249 UGX per person-day among Ugandan coffee farmers; substantially lower than the average of 10,000 UGX in our sample. In rural regions with high population densities and little non-agricultural employment, labour opportunity costs are low. Labour is thus best invested in farming activities (Chiarella et al. 2023). The loss of non-agricultural employment due to national COVID-19 regulation in our case study lead to low opportunity costs for household labour.

Future research could contribute to building an evidence base for smallholder farmers with labour-intensive cash crops, guiding their decisions on whether investing in SI is ‘worth it’. This involves calculating cost-benefit ratios for specific farming practices, such as alternative PDM. Such analyses require a comprehensive evaluation of labour inputs with more frequent data collection on labour and inputs to reduce recall bias, such as Dahlin and Rusinamhodzi (2019). As a note of caution, we cannot necessarily expect that the effects of changes in labour are symmetrical, i.e. that the effects of decreasing labour availability for farms that already have an established set of management practices are necessarily the converse of the effects of increasing labour availability. This deserves further research.

## Conclusions and recommendations

We evaluated empirically the importance of labour availability for a sustainable intensification (SI) of labour-intensive cash crops in smallholder farming systems, exemplified by pest and disease management. More specifically, this study represents an original, rigorous analysis of the relationship between changes in household labour availability and pesticide use on smallholder cocoa farms, mediated by the use of alternative pest and disease management practices. We selected a cocoa case study and made use of a unique quasi-experimental setting, in which household labour availability increased due to national COVID-19 restrictions in Uganda. Comparing data for the years 2019, just prior to the COVID-19 outbreak, and 2021 showed that household labour availability increased, while hired labour decreased significantly within a sample of 194 cocoa farms in Mukono district of Central Uganda. Our results showed that increased household labour was first used to satisfy basic food needs, but was also invested in cocoa production. Farms with increased household labour availability in 2021 compared to 2019 augmented their labour investments in cocoa production and alternative pest and disease management significantly more often than farms without increased labour availability. While our quantitative results only allow for a cautious conclusion that increased household labour availability influenced the adoption of alternative pest and disease management practices, they did confirm the important role of specific practices, namely phytosanitary measures and farm-made concoctions in cocoa, associated with a reduction in synthetic pesticide use on cocoa farms.

The adoption of alternative PDM practices represents an important pillar for the reduction or elimination of synthetic pesticides in labour-intensive cash crops in smallholder systems. It therefore represents one pathway towards SI. Future interventions for promoting sustainable farming and intensification must account for labour as an important adoption barrier (Abdulai et al. 2020). One approach is to reduce labour demand through mechanisation, such as the use of cocoa pod breaking machines to reduce post-harvest labour and injuries or motorised weed trimmers. Their uptake remains low, potentially due to high investment costs. Smallholder farmers need low-risk options with short-term returns on investment (Vanlauwe et al. 2014).

Alternatively, supporting and incentivising labour investments in sustainable practices can be done through economic mechanisms (Martínez & Martínez Pachón, 2021). This could lower the opportunity cost for farmers engaging in sustainable agriculture and potentially yield social-environmental co-benefits, a rare outcome of SI in low-income countries (Rasmussen et al. 2018). Sustainability certification, such as organic, in theory provides the economic incentives to eliminate the use of (highly toxic) synthetic pesticides through a price premium. In this study, this premium had not yet been paid to farmers and it remains to be seen if premiums are sufficiently high to motivate farmers to not only reduce pesticide use but also to invest their limited household labour in alternative pest and disease management practices. It is unlikely that sustainability certification is adequate and sufficient to drive a transformation towards sustainable agriculture (Meemken et al. 2021).

There are growing calls for public policy alongside private initiatives to tackle pressing issues (Lambin & Thorlakson 2018), such as the overuse of (highly toxic) synthetic pesticides. This could include incentives for alternative practices through payments for ecosystem services and taxes on highly toxic synthetic pesticides to discourage their use. Factors like knowledge of various practices (Adolph et al. 2020), trust in and perceived benefits of practices (de Oca Munguia & Llewellyn 2020), and membership in farmer organisations (Adolph et al. 2020; Meijer et al. 2015) also influence adoption of SI in smallholder cash crop production systems.

## Supplementary Information

Below is the link to the electronic supplementary material.

Supplementary file1 (DOCX 22 KB)

## Footnotes

1 Concoctions refer to farm-made natural inputs for PDM, e.g. fermented mix of urine, ash, and tobacco leaves.

2 Pesticide action network (PAN) created ‘bad actors’ to identify the ‘most toxic’ pesticides, which are at least one of the following: Known or probable carcinogens, reproductive or developmental toxicants, neurotoxic cholinesterase inhibitors, known groundwater contaminants, known high acute toxicity.

3 Highly hazardous pesticides ‘cause disproportionate harm to environment and human health including: severe environmental hazards, high acute and chronic toxicity’ (UNEP, n.d.)
