# Supplementary material for: The role of household labour for sustainable intensification in smallholder systems: a case study in cocoa farming systems
Source: Reg Environ Change. 2024 May 17;24(2):83. doi: 10.1007/s10113-024-02243-2 (PMC11608173; doi:10.1007/s10113-024-02243-2)
Supplement: Supplementary file 1 — Supplementary file1 (DOCX 22 KB) [file 10113_2024_2243_MOESM1_ESM.docx]

**Supplemental Material**

Supplementary material 1: Overview of mean (sd) percentages of crop types of total farm area in 2019 (partial overlap exists for short-cycle crops due to two cropping seasons)

| **Crop type** | **Mean (sd) percentage of farm area** |
| --- | --- |
| Cocoa | 17.35% (12.39%) |
| Coffee | 13.67% (11.38%) |
| Fallow | 13.08% (11.02%) |
| Grasses (elephant grass, grassland, sugar cane) | 12.82% (10.72%) |
| Grains (maize, rice, etc.) | 10.69% (9.65%) |
| Vegetables (tomato, cabbage, pumpkin, etc.) | 9.75% (6.83%) |
| Trees (eucalyptus, umbrella tree, etc.) | 8.22% (9.03%) |
| Bananas (sweet banana, plantain, etc.) | 7.79% (6.46%) |
| Roots/tubers (sweet potato, yam, cassava, etc.) | 7.73% (6.43%) |
| Pulses (ground nut, soy bean, etc.) | 5.81% (4.71%) |
| Spices (vanilla, cinnamon, etc.) | 4.61% (5.25%) |
| Fruits (avocado, jackfruit, orange, etc.) | 2.98% (3.63%) |

Supplementary material 2: Overview of active ingredients used by >5% of sampled farms in 2019 and/or 2021.

| Active ingredient | Type | Share farms 2019 | Share farms 2021 | ∆ | PAN Bad Actor | Highly hazardous pesticide |
| --- | --- | --- | --- | --- | --- | --- |
| Cypermethrin | Insecticide | 43% | 42% | +1% | No | Yes |
| Glyphosate | Herbicide | 39% | 26% | -13% | Yes | No |
| Profenofos | Insecticide | 15% | 20% | +5% | Yes | Yes |
| Chlorpyrifos | Insecticide | 10% | 4% | -6% | Yes | Yes |
| 2,4-D Amine | Herbicide | 8% | 6% | -2% | No | No |
| Lambda-Cyhalothrin | Insecticide | 6% | 21% | +15% | No | Yes |
| Mancozeb | Fungicide | 6% | 11% | +5% | Yes | Yes |
| Abamectin | Insecticide | 5% | 7% | +2% | Yes | Yes |
| Acetamiprid | Insecticide | 5% | 7% | +2% | No | No |
| Malathion | Insecticide | 3% | 6% | +3% | Yes | Yes |
| Thiamethoxam | Insecticide | 4% | 21% | +17% | No | Yes |

Supplementary material 3: Additional information presented as n (percentage) of the farmer sample

| Indicator | Total sample (*n*=204 in 2019 and *n*=193 in 2021) |
| --- | --- |
| Pest pressure in 2021 was perceived lower than in previous years (1/0) | 113 (58.2%) |
| National and international remittances received prior to Covid-19 (1/0) | 47 (24.2%) |
| Reduction in remittances since start of Covid-19 (1/0) | 28 (59.6% of farmers with remittances) |
| Application of biopesticides (1/0) | 1 (0.5% in 2019) / 2 (1% in 2021) |
| Input shortages due to national Covid-19 restrictions (1/0) | 13 (7%) |
| Covid-19 infections within household (1/0) | 47 (24%) |
| Longer absences from farm work due to Covid-19 infection (1/0) | 4 (2%) |

Supplementary material 4: Overview of pesticide need statements with 5-point Likert scale response options used to calculate the pesticide need index

| 1.Using pesticides increases farm profits |
| --- |
| 2.If I do not use pesticides, my crop will be lost |
| 3.Cocoa production without pesticides is impossible |
| 4.The easiest and best method of pest control is spraying synthetic pesticides |
| 5.Synthetic pesticides help in fighting pests and diseases |
| 6.Organic agriculture is not a good alternative to conventional agriculture |
| 7.Eating foods sprayed with pesticides is harmless for my family |

Supplementary material 5: Mean (sd) and n (percentage) for key characteristics of farms with more as well as same/less household labour availability in 2021 compared to 2019

|  | Same/less household labour availability (n=66) | Increased household labour availability (n=128) | *p*-value |
| --- | --- | --- | --- |
| Change active ingredient pesticide (kg / ha) | 1.11 (4.41) | 0.42 (4.13) | 0.368 |
| Change expend. synthetic pesticides (1000 UGX / ha) | 33.76 (116.93) | 5.76 (139.68) | 0.303 |
| Farm size (ha) | 2.42 (1.95) | 3.07 (3.43) | 0.191 |
| Male farm manager (%) | 52 (78.8%) | 81 (63.3%) | 0.028 |
| Age farm manager (years) | 51.49 (13.47) | 53.26 (12.00) | 0.341 |
| Training participation since 2020 (%) | 35 (53.0%) | 70 (54.7%) | 0.826 |
| Cocoa yields 2019 (tons / ha) | 0.36 (0.56) | 0.36 (0.40) | 0.269 |
| Cocoa yields 2021 (tons / ha) | 0.55 (0.55) | 0.68 (0.72) | 0.159 |
| Cocoa revenue 2019 (1M UGX) | 2.39 (4.42) | 2.40 (3.21) | 0.034 |
| Cocoa revenue 2021 (1M UGX) | 2.15 (4.00) | 2.74 (4.37) | 0.016 |
| Pruning cocoa 2019 (%) | 19 (28.8%) | 41 (32.0%) | 0.643 |
| Pruning cocoa 2021 (%) | 55 (83.3%) | 107 (83.6%) | 0.963 |
| Concoctions use in cocoa 2019 (%) | 15 (22.7%) | 30 (23.4%) | 0.912 |
| Concoctions use in cocoa 2021 (%) | 17 (25.8%) | 58 (45.3%) | 0.008 |
| Phytosanitary measures in cocoa 2019 (%) | 23 (34.8%) | 53 (41.4%) | 0.375 |
| Phytosanitary measures in cocoa 2021 (%) | 36 (54.5%) | 86 (67.2%) | 0.084 |
| Notes: Statistical comparison between years using McNemar's Chi-squared test with continuity correction for binary variables; Wilcoxon rank sum test with continuity correction for continuous variables | | | |

Supplementary material 6: Results of the ordinary least square (OLS) models testing the relationship between changes in household labour availability and changes in pesticide quantities and expenditures.

|  | Change in pesticide quantities 2021-2019 (kg active ingredient / ha) ^a^ | | Change in pesticide expenditures 2021-2019 (1000 UGX / ha) ^a^ | |
| --- | --- | --- | --- | --- |
|  | Coef. (robust SE) | *p*-value | Coef. (robust SE) | *p*-value |
| Change household labour (1000 h/ha) ^a^ | -0.045 (0.052) | 0.392 | -0.038 (0.250) | 0.881 |
| Change hired labour (1000 h/ha) ^a^ | -0.226 (0.144) | 0.118 | -0.577 (0.627) | 0.358 |
| Male farm manager (1/0) | -0.229 (0.119) | 0.056 | -0.845 (0.553) | 0.127 |
| Age farm manager (yrs) | 0.004 (0.005) | 0.417 | 0.013 (0.022) | 0.563 |
| Farming experience (yrs) | -0.002 (0.004) | 0.531 | -0.032 (0.019) | 0.082 |
| Commercial vegetable production (1/0) ^b^ | 1.709*** (0.456) | 0.0002 | 4.851** (1.521) | 0.002 |
| Pruning cocoa (1/0) ^b^ | -0.459** (0.169) | 0.007 | -1.368 (0.714) | 0.056 |
| Concoctions use (1/0) ^b^ | -0.225 (0.121) | 0.064 | -1.181* (0.541) | 0.03 |
| Phytosanitary measures cocoa (1/0) ^b^ | -0.019 (0.128) | 0.884 | -0.288 (0.607) | 0.635 |
| Training since 2020 (1/0) | -0.242 (0.125) | 0.053 | -0.149 (0.543) | 0.784 |
| Pesticide price (M UGX/kg AI) ^b^ | -1.021* (0.449) | 0.023 | 6.877** (2.323) | 0.004 |
| Cocoa trees <3 years (% of trees) | -0.061 (0.194) | 0.753 | -0.562 (0.892) | 0.529 |
| Importance cocoa (% farm revenues) ^b^ | 0.019 (0.206) | 0.926 | 1.197 (1.054) | 0.256 |
| Importance vegetables (% farm revenues) ^b^ | -1.337 (1.592) | 0.402 | -5.830 (4.728) | 0.218 |
| Farm size (ha) ^a^ | -0.117 (0.092) | 0.205 | -0.185 (0.418) | 0.658 |
| Constant | 0.826* (0.397) | 0.038 | 2.193 (1.600) | 0.171 |
| Observations | 194 |  | 194 |  |
| R^2^ | 0.298 |  | 0.192 |  |
| Adjusted R^2^ | 0.239 |  | 0.124 |  |
| Residual Std. Error (df = 178) | 0.807 |  | 3.539 |  |
| F Statistic (df = 15; 178) | 5.036*** |  | 2.823*** |  |
| Note: *p<0.05; **p<0.01; ***p<0.001 | | | | |

**^a^**IHS transformed

^b^2021 data
